# Online sale of small turtles circumvents public health regulations in the United States

**DOI:** 10.1371/journal.pone.0278443

**Published:** 2022-12-21

**Authors:** Lauren E. Montague, Juliana M. Marcotrigiano, Niamh E. Keane, Hannah E. Marquardt, Jennifer A. Sevin, Nancy E. Karraker

**Affiliations:** 1 Department of Natural Resources Science, University of Rhode Island, Kingston, Rhode Island, United States of America; 2 Department of Biology, University of Richmond, Richmond, Virginia, United States of America; Texas State University, UNITED STATES

## Abstract

In the United States (U.S.), pet turtles have been associated with outbreaks of salmonellosis, a serious and sometimes-fatal intestinal illness caused by *Salmonella* bacteria, with nearly 300,000 people being infected in some years. Children are particularly susceptible because of their propensity to put items, including small turtles, in their mouths. In 1975, a U.S. federal regulation prohibited the sale of turtles <4 inches (101.6 mm) in size, except for the purposes of export, scientific, or educational purposes. This regulation was established to reduce the incidence of salmonellosis, particularly in small children. Previous research has not evaluated the availability of turtles <4 inches in size on websites selling wildlife. We monitored 16 websites in 2021 and quantified listings of small turtles. We determined whether information on *Salmonella*, the 1975 federal regulation, or related state regulations were provided on the websites and determined legality of sales of small turtles by state regulations. We found that all 16 websites openly advertised and sold turtles <4 inches in size, but only half of these websites provided information about *Salmonella* and/or the federal regulation. These websites required buyers to confirm that they were not purchasing a turtle as a pet, thereby putting the onus on the consumer to adhere to the regulation. We documented 515 listings of turtles <4 inches in size, including 47 species and one hybrid. Our study has demonstrated that internet sales of small turtles currently represent part of the thriving online pet trade in the U.S. Enforcement of the federal regulation faces jurisdictional challenges in most states. Therefore, we recommend continued public education campaigns by public health agencies in the U.S. to help reduce the risk that pet turtle ownership presents.

## Introduction

In the 1960s and 1970s, hatchling turtles became increasingly popular pets in the United States. By the early 1970s, about 4% of households in the U.S. owned at least one turtle, and about 15 million hatchling turtles, primarily red-eared sliders (*Trachemys scripta elegans*), were being sold annually [1]. As hatchlings, red-eared sliders are about the size of a U.S. quarter (24.3 mm), and their size, coupled with a lime green shell and red stripes on the sides of their heads, made them popular and highly desired as pets. During the same period, the Centers for Disease Control and Prevention was documenting salmonellosis, an intestinal disease caused by *Salmonella* bacteria, cases in the public, and it was estimated that approximately 280,000 cases per year were attributable to turtle ownership [1]. Small children, in particular, were contracting salmonellosis because of poor hygiene after handling turtles and putting small turtles in their mouths. Salmonellosis can cause severe illness and death, particularly in young children, the elderly, and those with compromised immune systems [2]. In the 1970s, a public health education program focused on turtle-associated salmonellosis was launched by the Centers for Disease Control and Prevention [3], but this apparently did little to stem the number of infections. As a result, the U.S. Food and Drug Administration formulated a regulation [4] that prohibited sale in the U.S. of live turtles with a carapace (upper shell) length of <4 inches (101.6 mm), with the exception of turtles “intended for bona fide scientific, educational, and exhibition purposes, other than use as pets.” This size restriction was included in the regulation as it was determined that because, “turtles with a carapace, i.e., upper shell, length of less than 4 inches are the more common pet varieties purchased for or by children, the ban should not be extended to include turtles whose carapace is larger than 4 inches [4].” The regulation permits export of turtles with a shell length of <4 inches in size from the U.S. Although no records on early enforcement of the regulation are available, the number of turtle-associated salmonellosis cases in children declined by 77% between 1972 and 1976 [3], reinforcing the combined value of education and regulation to protect public health.

Up to the establishment of the regulation, the most popular turtles were red-eared sliders which were sold via brick-and-mortar pet stores and by mail-order [1]. The emergence of the internet, and subsequent ability to shop online, in the 2000s changed the nature of the pet trade [5]. Turtles and tortoises became more easily attainable by anyone using the internet, regardless of their proximity to the seller. Customers and sellers are also able to remain anonymous through transactions, providing protection and confidentiality by hiding locations and identities [6]. Although some degree of anonymity was possible through mail order, sellers’ business addresses were apparent and buyers’ names and home addresses were associated with purchases.

Although the 1975 Food and Drug Administration regulation prohibiting the sale of turtles <4 inches in size was established nearly 50 years ago to protect the public from salmonellosis, children continue to become infected with *Salmonella* through contact with small turtles [7]. The availability of small turtles for sale on the internet, in contravention of the federal regulation, has not been previously assessed. Therefore, the objectives of our study were to: (1) quantify the availability of turtles <4 inches in size on websites selling turtles, (2) document information provided by these websites on *Salmonella*, the 1975 Food and Drug Administration regulation, and relevant state regulations; and (3) determine whether states in which websites are registered/operate have separate regulations on the sale of small turtles.

## Materials and methods

We identified websites that sold turtles <4 inches in size using the Google search engine and our search terms included: “baby turtles for sale,” “turtles for sale,” “exotic reptiles for sale,” and variations of these search terms. We collected data during two different time periods, February to March 2021 and October to November 2021, with the intention of distributing monitoring effort at different times of the year. Our original goal was to survey 10 websites during each monitoring period, but we were only able to find eight websites with small turtles for sale at the outset of each period. Thus, during each of the two periods, we monitored eight different websites for a total of 16 websites over the course of the study. We collected data only from listings that clearly stated the size of the turtle, in order to confirm that sales on the websites were in violation of the federal regulation. If a website stated that a turtle was <4 inches in size or 3–5 inches in size, we included it in data collection. We ensured that turtles advertised on the website were actually available for sale by adding them to our ‘shopping cart.’ We did not include marine turtles within the families *Dermochelyidae* and *Cheloniidae*, because species in these families are exempt from the 1975 law.

For each listing that advertised turtles of <4 inches in size, we recorded common name, scientific name (if provided), and advertised size. We determined the scientific name for the species being sold if it was not provided. As the common names and scientific names used by particular websites varied, we standardized scientific names for listings [8]. We documented details from the websites which pertained to regulations concerning the sale of turtles <4 inches in size, including restrictions on shipment to particular states or turtles advertised as being sold for scientific or educational purposes.

Using the data we collected, we determined the numbers of listings of turtles <4 inches in size on each website and documented which websites provided information on the risk of *Salmonella*, the 1975 FDA regulation, or related state regulations.

## Results

All 16 websites we evaluated openly advertised and sold turtles <4 inches in size. During the study period, we documented 515 listings of turtles advertised as <4 inches in size. Of these, 44% were advertised as being ≤2 inches in size. As not all websites indicated how many individuals were available, we were unable to quantify the total numbers of undersized turtles being sold. As our goal was to determine if websites were selling small turtles in contravention of the 1975 regulation and not to target particular businesses for scrutiny, we chose not to reveal the names of the websites we monitored and assigned a number for reference to the business instead.

Warnings about the risk of *Salmonella* from handling turtles or references to the 1975 regulation appeared on 50% of websites, either with individual listings of turtles for sale or in other places on the website. Two websites included a statement warning about the risk of *Salmonella* with each individual listing of turtles <4 inches in size (S1 Table). For the website associated with a business in Texas, a statement was included for 33% of 49 total listings. For a single business that operates out of California, Idaho, and Texas, a warning about *Salmonella* appeared with 100% of 15 listings. Six websites provided information on a terms and conditions page or in the information on shipping. Two websites provided a warning about the risk of *Salmonella* and included language asking the buyer to confirm that it is legal for the buyer to own or possess a turtle in their state for bona fide scientific, educational, or export purposes. Four other websites (CA/ID/TX, FL, NJ) included no information about *Salmonella* but indicated that turtles <4 inches in size were being sold solely for scientific, educational, and/or export purposes. One of these websites (NJ) included language directly from the 1975 FDA regulation, and two of these websites (CA/ID/TX, FL) indicated that turtles <4 inches in size were not being sold for use as pets.

We found that listings for turtles <4 inches in size included 47 species and one hybrid (S1 Table). Of these, the most common species advertised were sliders (17%; *Trachemys scripta*), followed by Eastern painted turtles (7%; *Chrysemys picta*), and common musk turtles (6%; *Sternotherus odoratus*).

Of 16 websites monitored, we determined that their business headquarters were located in or they operated in eight states (Table 1). Of these websites, 56% were located in Florida. We determined that three of these websites operate in states (California, Michigan, New Jersey) with state-level wildlife regulations that explicitly reinforce the federal ban and prohibit the sale of turtles <4 inches in size (Table 1). Only two of 16 websites provided information (totaling 22% of listings) indicating that sales of particular species were restricted for particular states (Table 2).

**Table 1:** States in which headquarters or operations centers are located for 16 websites selling turtles <4 inches in size and state regulations applicable to these sales [9]. One business operated from three states (CA, ID, TX); all others operated from one state.

State	Number of businesses	Regulatory language	Regulatory code
California	2	Unlawful to import, sell or offer for sale or distribution to the public any live turtle(s) with a carapace length of less than 4 inches.	Cal. Code Regs, tit. 17, § 2612.1
Florida	9	None	
Idaho	1	None	
New Jersey	1	(a) Viable turtle eggs and live turtles with a carapace length of less than four inches shall not be sold, held for sale, or offered for any other type of commercial or public distribution. (b) Live turtles of carapace length of four inches or greater shall not be sold or in any way distributed within the state unless the entity seeking to sell or distribute the turtles warrants to the satisfaction of the Department of Health and Senior Services that each shipment of turtles is free from Salmonella contamination.	N.J. Admin. Code 8:23–2.1
Michigan	1	A person shall not sell or distribute in the state viable turtle eggs or live turtles with a carapace length of less than 4 inches unless he or she provides the purchaser with the health advisory sheet described in section 3.	Mich. Comp. Laws § 287.312
Nevada	1	None	
Texas	3	None	

**Table 2:** Information provided on two websites regarding restricted sales or shipping to particular states for turtles <4 inches in size. Information provided for business number 16 (Florida) for sliders (*Trachemys scripta*, all subspecies combined) changed among listings and over the course of our surveys; all information that appeared related to *T*. *scripta* for business number 16 during our surveys is provided below.

Business number, state	Species	Percent of listings providing information	Information provided
4, FL	*Chelydra serpentina*	60	No California sales
4, FL	*Trachemys scripta*	14	No Florida sales
16, FL	*Centrochelys sulcata*	100	Restricted states: OR, WA, HI
	*Chelonoidis carbonarius*	100	
	*Cuora amboinensis*	100	
	*Emydura subglobosa*	100	
	*Indotestudo elongata*	100	
	*Mauremys annamensis*	100	
	*Mauremys nigricans*	100	
	*Mauremys reevesii*	100	
	*Pseudemys peninsularis*	100	
	*Trachemys grayi*	100	
	*Trachemys ornata*	100	
16, FL	*Chrysemys picta*	25	Restricted states: OR, AZ, WA, HI
16, FL	*Clemmys guttata*	100	Restricted states: HI, GA, ME, NH, VT, MA, RI, CT, NY, NJ, DE, MD, VA, WV, PA, OH, OR, MI, WA, IN, NC, SC, IL
16, FL	*Glyptemys insculpta*	100	Restricted states: ME, NH, VT, MA, RI, CT, NY, NJ, DE, MD, VA, WV, PA, OH, MI, WI, IA, WA, OR, HI
16, FL	*Sternotherus odoratus*	100	Restricted states: OR, NY, RI, WA, HI
16, FL	*Trachemys scripta*	8	No shipping to VA or FL; Restricted states: VA, OR, MA, WA, HI
16, FL	*Trachemys scripta*	11	Not allowed to ship to VA or FL; Restricted states: OR, MA, WA, HI
16, FL	*Trachemys scripta*	43	Restricted states: VA, FL, OR, GA, MA, HI, WA
16, FL	*Trachemys scripta*	5	Restricted states: OR, GA, MA, NY, WA, HI
16, FL	*Trachemys scripta*	6	Restricted states: OR, MA, WA, HI
16, FL	*Trachemys scripta*	16	Restricted states: OR, WA, HI

Prices of turtles <4 inches in size advertised on these websites ranged broadly. We documented the lowest prices (USD) for red-eared sliders and common snapping turtles (*Chelydra serpentina*), both advertised at their lowest prices for $6 each. The most expensive listings were for Indian star tortoises (*Geochelone elegans*; $800–2500) and ‘ivory’ sulcata tortoises (*Geochelone sulcata*; $995–1295). Websites appeared to be catering to particular clientele based on price and species’ rarity. Listings during our study were distributed as: 12% of websites advertised only inexpensive listings ($15–60), 69% of websites advertised listings spanning a broad price range (<$50–2,500), and 19% of websites advertised only expensive listings ($205–2,500).

## Discussion

Over the past 20 years, the internet has become a remarkably effective tool for both sellers and buyers of illegally traded wildlife, including turtles [10, 11]. Websites selling wild animals allow sellers and buyers to remain anonymous, thereby facilitating trade that would have been riskier to each in a brick-and-mortar pet shop [12]. Given the current-day profusion of websites selling turtles, it is challenging to monitor the species and sizes of turtles being sold and, thus, enforce the federal and state regulations that prohibit the sale of turtles <4 inches in size. This allows sellers to bypass regulations while making a living in the wildlife trade and permits buyers to intentionally or unwittingly purchase small turtles in contravention of regulations. Assessing the scale of this issue is important, particularly given that in the U.S. turtles are the most common reptiles kept as pets [13].

The 1975 regulation focused on the important public health goal of protecting people, and especially children, from accidentally becoming infected with *Salmonella*, but enforcement of the regulation is complicated by jurisdictional issues and *Salmonella* outbreaks associated with small turtles still occur. In fact, between August 2020 and July 2021, an outbreak of *Salmonella* infections was investigated by the Centers for Disease Control and Prevention [7]. At least 87 people from 20 states and the District of Columbia became infected. Ages of infected people ranged from less than one year old to 85 years old, with a median age of six years old, and 45% of infected persons were less than five years of age. Of 74 people for whom health care information was reported, 43% were hospitalized and one person died. Of 63 people who provided information, 76% reported coming into contact with a pet turtle before becoming infected, and 82% of 33 people who reported turtle size indicated that their turtle was <4 inches in size [7]. Between January and August 2022, a *Salmonella* outbreak that spanned 14 U.S. states was associated with turtles <4 inches in size purchased from an online retailer that specializes in turtles [7].

Eighteen states in the U.S. prohibit the sale of small turtles [9]. Of these states, 14 have specific regulations banning the sale of turtles <4 inches in size, three explicitly acknowledge and adhere to the federal standard in their code of regulations, and one state bans the sale of turtles <6 inches in size [9]. State law enforcement officers may not have the jurisdictional authority to enforce the federal regulation if their state does not explicitly indicate adherence to it in their code of regulations. Of the 16 websites selling turtles we assessed, two were selling small turtles in violation of the regulations of the states–California, New Jersey–in which these businesses were located. Notably, two monitored websites, associated with businesses registered to a single individual, were no longer publicly available after we completed data collection. These two websites had received 235 complaints to the Better Business Bureau largely regarding payment having been made but customers never having received their purchase or having received dead or ill animals. Although we documented 69 listings for turtles <4 inches in size on these websites, it is more likely that these two websites closed because of fraudulent business practices rather than for enforcement action related to violation of the 1975 regulation.

Half of the websites we monitored provided information pertaining to the risk of *Salmonella* and/or referencing the 1975 regulation directly with the listings, in the terms and conditions, or in the shipping information. In essence, by agreeing to the terms and conditions on these websites the buyer is confirming that they are not purchasing a turtle <4 inches in size as a pet and that the turtle is being used only for educational or scientific purposes. The sellers are attempting to place responsibility for adherence to the federal regulation and any related state regulations on the consumer. However, the regulations prohibit the sale of turtles <4 inches in size, which means that the responsibility is that of the seller. Further, details provided in the federal regulation make it clear that simply stating that a turtle may only be bought for educational or scientific purposes contravenes the intent of the regulation regarding these exceptions. To wit, “The Commissioner concludes that this exception is reasonable and will not present a public health hazard since the scope of the exception is limited to a specific segment of society consisting of experts in the field who are fully aware of the contamination problems associated with turtles and the necessary precautions required to prevent such contamination. While turtle fanciers may be similarly competent to handle turtles safely, no comment has suggested, and the Commissioner has been unable to ascertain an enforceable exemption that would require a business to restrict its sales only to such qualified persons [4].” In addition, “The Commissioner concludes that these exceptions would not constitute a significant hazard to public health due to the limited accessibility of the general public to turtles used for these particular purposes [4],” indicating that the intent of the regulation was that the general public would be unable to purchase turtles <4 inches in size. Thus, regardless of any information provided to consumers, by suggesting that turtles may be purchased for educational or scientific purposes, sellers are violating the federal regulation and state regulations in some cases.

We documented a wide range of prices for advertised turtles <4 inches in size on these websites and prices appeared to be associated with rarity, as has been found in internet traded mammals [14]. Common species, such as red-eared sliders and common snapping turtles, were advertised at lower prices, and endangered species, such as sulcata tortoises and Indian star tortoises, were advertised at much higher prices. It appeared that these websites had targeted audiences. Only 19% of websites appeared to be advertising turtles specifically for collectors or advanced hobbyists with prices ≤$200 and ranging up to $2,500. Most (69%) websites advertised small turtles for sale across a wide range of prices, demonstrating that a broad span of interests, from purchasing an inexpensive family pet to obtaining a highly coveted rare species as a collector or more advanced hobbyist, can be accommodated by most of these websites. The range in prices on these websites suggest that website owners know there is a large market with high variability in customers to whom they can sell. About 12% of websites appeared to be focused solely on advertising small turtles as family pets or first-time pets with prices ≤$60. Thus, >80% of websites we monitored sell small turtles priced for a broad range of consumers including those in households with small children.

The U.S. state of Louisiana played an important role in the production of hatchling pet turtles (primarily red-eared sliders), with a turtle farming industry that was well-established by the 1950s [15]. By 1969, Louisiana had 75 pet turtle farming operations with an estimated production of 15 million hatchling turtles annually [16]. On farms in Louisiana and other states, turtles are held and bred in large ponds, wherein the combination of standing water and dead fish provided as food created an optimal environment for the growth of *Salmonella* [17]. Turtle farming in the U.S. was impacted by passage of the 1975 regulation by prohibiting sales of small turtles [16]. Although this regulation did not prohibit the international export of turtles <4 inches in size, many U.S. turtle farms closed or scaled back operations as the turtle farming industry in China began to grow [15]. However, the U.S. industry exhibited some recovery beginning in the late 1980s. Pet turtle sales from farms in Louisiana increased from about 4 million turtles per year in 1986 to just above 8 million turtles per year in 1997, and these farms supplied 85–90% turtles for the global market in 1997 [15].

In 2006, the Independent Turtle Farmers of Louisiana petitioned the U.S. Food and Drug Administration to lift the ban on the sale of turtles <4 inches in size, in part because of laboratory research and field application of methods to reduce the incidence of *Salmonella* in farmed turtles [18]. The Food and Drug Administration rejected the petition citing a lack of consistent evidence that *Salmonella*-free turtles could be produced and that turtles would not become contaminated during shipment. This led to a lawsuit [18] in which several issues with the Food and Drug Administration regulation were raised. Ultimately the court determined that further proceedings were needed at the Food and Drug Administration level, rather than vacating the regulation [18]. Although the regulation is still in place, between 2006–2014, 15 turtle-associated salmonellosis outbreaks, resulting in 921 illnesses, 156 hospitalizations, and one death of a three week-old infant, were investigated by the Centers for Disease Control and Prevention [13]. Of these, the median age for individuals who became ill was <10 years of age. In 2015, four multi-state salmonellosis outbreaks linked to small turtles resulted in 143 ill people in the U.S., of which 45% were children aged <5 years in age [19]. These studies demonstrate that turtle-associated salmonellosis remains an important public health concern particularly with regard to children.

One positive but unintended consequence of the 1975 regulation and related state laws may have been the reduction in sales of red-eared sliders in the U.S., a turtle considered to be one of the 100 most detrimental invasive species in the world [20]. This species has been released into the wild and became established outside of its native in the U.S. However, just over 15% of the listings we document were for red-eared sliders indicating that these turtles can still be easily purchased on the internet and potentially be released to the wild when their owners lose interest or they become larger and more difficult to care for.

Limited research has investigated online sales of turtles, but researchers, particularly in China, are monitoring websites for sales of endangered turtles [10, 11] and invasive turtle species [21]. In New Zealand, the internet pet trade was monitored for sales of red-eared sliders as an indicator of invasive propagule pressure via the pet-release pathway [22]. A similar approach could be taken with regard to public health vulnerability associated with the sale of small turtles. We found that the availability of turtles <4 inches in size for purchase, the number of websites offering small turtles for sale, and the availability of salmonellosis information for consumers can be monitored on websites in the U.S. selling turtles. Information collected from these sites could serve as a tool to monitor the risk of turtle-associated salmonellosis outbreaks in the U.S.

## Conclusion

The sale of turtles <4 inches in size poses a public health risk by promoting the potential spread of *Salmonella* to humans, especially the young, the elderly, and those with compromised immune systems. Despite passage of the Food and Drug Administration regulation nearly 50 years ago prohibiting the sale of these turtles, our research has demonstrated that internet sales of small turtles currently represent part of the thriving online pet trade in the U.S. Enforcement of the regulation is likely done in an ad hoc manner in brick-and-mortar pet stores, but there are jurisdictional challenges to enforcement of this federal regulation by state agents in most states. In addition, law enforcement agents at the state and federal levels may feel that they have more important issues regarding internet-based wildlife sales, including monitoring sales of endangered species or potentially injurious species, such as big cats or primates. However, salmonellosis outbreaks continue to occur in the U.S. and are regularly attributed to pet turtles, which represent a more expansive public health concern than that posed by potentially injurious animals. State-level regulations restricting the sale of turtles <4 inches in size would provide state law enforcement agents increased ability to stop these sales and hold business owners responsible, particularly during periods with significant salmonellosis outbreaks. It is likely that most members of the public are not aware that the sale of turtles <4 inches in size as pets is illegal in the U.S. and that pet turtles are regularly associated with salmonellosis outbreaks. Public education campaigns by the U.S. Food and Drug Administration, Centers for Disease Control and Prevention, and state public health agencies that explain the 1975 regulation, related state regulations, and the important public health reasons for the regulations would allow consumers to make informed decisions about purchasing turtles as pets.

## Supporting information

S1 Table Turtles ≤4 inches in size being sold in violation of U.S. regulation. Data collected on turtles ≤4 inches in size for sale on 16 websites in February-March and October-November 2021.
(XLSX)
